# The Effect of Hydrofluoric Acid Surface Treatment on the Cyclic Fatigue Resistance of K3 NiTi Instruments

**DOI:** 10.1155/2017/3189729

**Published:** 2017-05-02

**Authors:** Kee-Yeon Kum, Seok Woo Chang

**Affiliations:** ^1^Department of Conservative Dentistry, Seoul National University Dental Hospital, Seoul Dental Hospital for Disabled, Dental Research Institute, Seoul National University School of Dentistry, Seoul, Republic of Korea; ^2^Department of Conservative Dentistry, School of Dentistry, Kyung Hee University, Seoul, Republic of Korea

## Abstract

The aim of this study was to investigate the effect of 50% hydrofluoric acid (HF) surface treatment on the cyclic fatigue resistance (CFR) of K3 NiTi instruments. Twenty as-received and twenty HF-treated K3 NiTi instruments were compared in CFR. The surface texture and fracture surface of two instrument groups were examined with a scanning electron microscope (SEM). Additionally, any change of Ni and Ti composition from both instrument groups was investigated using energy dispersive spectrometry. The results were analyzed with *t*-test. The HF-treated K3 group showed statistically higher cyclic fatigue resistance than as-received K3 group (*P* < 0.05). HF-treated K3 instruments showed smoother and rounded surface compared to as-received K3 under SEM observation. The fracture surfaces of both groups showed typical patterns of cyclic fatigue fracture. There was no difference in surface Ni and Ti composition between two groups. HF treatment of K3 instruments smoothed the file surface and increased the cyclic fatigue resistance, while it had no effect on surface ion composition and the file fracture pattern.

## 1. Introduction

In modern endodontics, many clinicians use engine driven nickel-titanium (NiTi) rotary instruments, which makes root canal preparation more fast and effective [1]. However, accidental instrument separation is a concern for every clinician who uses NiTi rotary instruments in their practice. The two main fracture mechanisms of NiTi rotary instrument are cyclic fatigue fracture and torsional fracture [2]. Cyclic fatigue fracture has been reported to be affected by many factors such as manufacturing process [2], root canal geometry [3], instrumentation motion [4], cross-sectional configuration, rotational speed [5], and surface treatment method [6]. It is recently focused that surface defects such as microcracks and machining grooves, which were induced in machining process during manufacturing, are the stress concentration point where the initiation of file fracture begins to propagate [5, 7–9]. The grinding procedure across the grains of NiTi wire has been also suggested to deteriorate the mechanical strength of the instrument owing to the machining defects on instrument surface and the residual stress within the internal structure [10–12]. Previous studies supported this postulation in that the ground file showed much lower cyclic fatigue resistance than twisted files [5]. Therefore, various surface treatment methods such as electropolishing [13], ion implantation [14], and chemical and physical vapor deposition [15, 16] have been tried for improving mechanical properties and reducing surface defects of ground NiTi rotary instruments. These methods were reported to be effective in improving the mechanical properties of NiTi rotary instruments, but there have not been firmly established consensus about the effectiveness of such treatment for improving cyclic fatigue resistance of NiTi rotary instruments, yet.

Acid treatment is widely used method during manufacturing process to chemically polish the surface of ground NiTi instruments. Hydrofluoric acid (HF), which is the ingredients of Kroll's reagent, is used for chemical polishing of ground NiTi alloy [17]. In spite of the surface treatment during manufacturing, previous studies clearly showed that the as-received ground NiTi rotary instruments have rough surface which include rolling marks, microcracks, and pitting under a scanning electron microscope (SEM) [5, 18]. In addition, our preliminary study showed that chemical polishing with higher concentrations of HF produced smoother surface of NiTi instruments compared with that of as-received NiTi instruments (unpublished data).

Therefore, in this study, we compared the cyclic fatigue resistances of K3 NiTi rotary instruments before and after surface treatment with HF. In addition, to visually examine any change induced in the instrument surface, the SEM images for as-received and HF-treated K3 instruments were investigated. Furthermore, EDS (energy dispersive spectrometry) analysis was carried out to investigate any change in surface ion composition of K3 files by HF surface treatment. Fractographic analysis of fracture surface was also performed under SEM.

## 2. Materials and Methods

### 2.1. Treatment of K3 NiTi Rotary Instruments with Hydrofluoric Acid

The NiTi rotary instruments used in this study were K3 with #30/06 taper (SybronEndo, Orange, CA). Twenty as-received K3 instruments were soaked in 50% HF for 2 minutes and washed with distilled water. Twenty as-received K3 instruments were included as a control group.

### 2.2. Cyclic Fatigue Resistance Test

**Figure 1:**
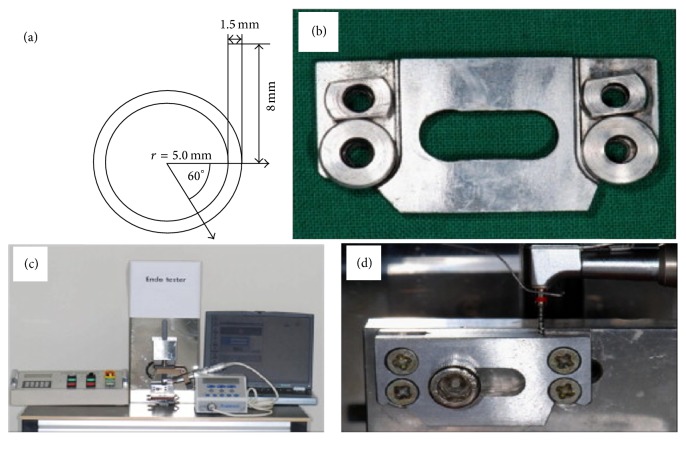
Cyclic fatigue resistance measuring equipment and diagram of an artificial canal. (a) Schematic diagram of artificial canal. (b) Metal blocks constituting artificial canal. (c) Setup of cyclic fatigue resistance measuring equipment. (d) Working NiTi instrument inside the artificial canal during experiment.

The cyclic fatigue resistance was measured with 20 as-received K3 instruments and 20 HF-treated K3 instruments. All the instruments had been previously examined using an OPMI dental surgical microscope (Zeiss, Oberkochen, Germany) for any sign of surface defect or deterioration. A fatigue tester (Denbotix, Bucheon, Korea) was used in this study. This equipment was designed to provide cyclic tensile and compressive stress on the tip area of the instrument, so that it simulates the conditions encountered in clinical situations [5] (Figure 1).

A stainless-steel artificial canal with 1.5 mm inner diameter, 8 mm length (straight portion), 60° angle of curvature, and 5 mm radius of curvature was used in this experiment. To simulate the up and down pecking motion, continuous 6 mm up and down motion was applied at 0.5 cycles per second. Each NiTi instrument was engaged on an electric torque-controlled motor (Aseptico, Woodinville, WA) with a 20 : 1 reduction handpiece and rotated at the speed recommended by manufacturer (300 rpm). During experiment, the artificial canal was filled with RC-prep (Premier Dental Products, Norristown, PA, USA) to minimize the friction and heat generation in artificial canal. When an instrument fractures in the artificial canal, the internal sensor detects the torque change on the canal wall and the electric motor automatically stops. Thereafter, the experimenter visually confirmed instrument fracture. The time elapsed until the instrument fracture was automatically recorded by a digital timer accurate to 0.1 seconds. The time to fracture was multiplied by the number of rotations per minute (rpm), to calculate the number of cycles to failure (NCF) for each NiTi instrument. Using the SPSS statistical package (Version 12, SPSS, Chicago, IL), the NCF of as-received and HF-treated samples were analyzed by one-way analysis of the variance (ANOVA) and Tukey post hoc test. *P* value less than 0.05 was considered to be statistically significant.

### 2.3. SEM Examination of As-Received and HF-Treated Surfaces of K3 Instruments

To investigate the effect of HF on the surface texture of K3 instruments, as-received and HF-treated instruments were examined with SEM (Hitachi S-4700, Tokyo, Japan) at a magnification of ×200 and ×10,000.

### 2.4. Comparison of Ni and Ti Composition in As-Received and HF-Treated K3 Files

To investigate the effect of HF on the change of Ni and Ti ion composition of K3 instrument, energy dispersive spectrometry (EDS) was carried out in as-received and HF-treated K3 instruments (*n* = 3).

### 2.5. Examination of Fracture Surfaces

After the cyclic fatigue fracture, the fracture surfaces were examined by SEM. Fractographic analysis of all fractured instruments was performed by SEM (Hitachi S-4700, Tokyo, Japan) at magnifications of ×5,000 to determine the modes or patterns of fracture.

## 3. Results

### 3.1. Cyclic Fatigue Resistance Test

The results of cyclic fatigue resistance test were shown in Table 1. The cyclic fatigue resistance in HF-treated K3 files was significantly higher than that of as-received K3 files (*P* < 0.05).

### 3.2. SEM Examination of Surfaces in As-Received and HF-Treated K3 Files

**Figure 2:**
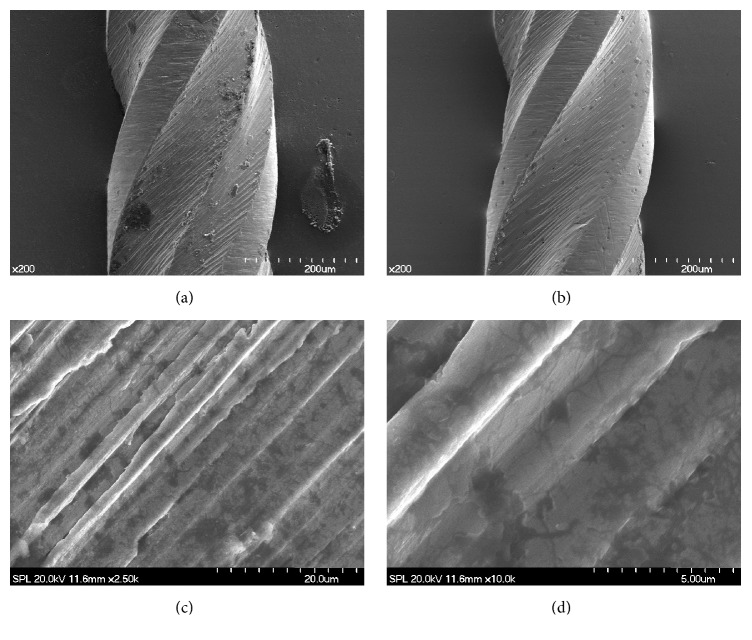
SEM images of the surfaces of as-received and HF-treated K3 instruments. As-received K3 files (a, c) revealed rough surfaces with metal roll-overs and machining marks. On the contrary, the HF-treated K3 (b, d) showed smoothening of rough surfaces. (a) As-received K3 instrument (×200). (b) HF-treated K3 instrument (×200). (c) As-received K3 instrument (×2,500). (d) HF-treated K3 instrument (×10,000). SEM: scanning electron microscope; HF: hydrofluoric acid.

SEM images of as-received K3 instruments clearly showed rough surfaces with metal roll-overs and machining marks (Figures 2(a) and 2(c)). On the contrary, the HF-treated K3 showed smoothening of rough surfaces (Figures 2(b) and 2(d)). It was shown that the flute surface of K3 instrument was smoothened and the sharp edges of milling mark were rounded.

### 3.3. Comparison of Ni and Ti Composition in As-Received and HF-Treated K3 Files

**Figure 3:**
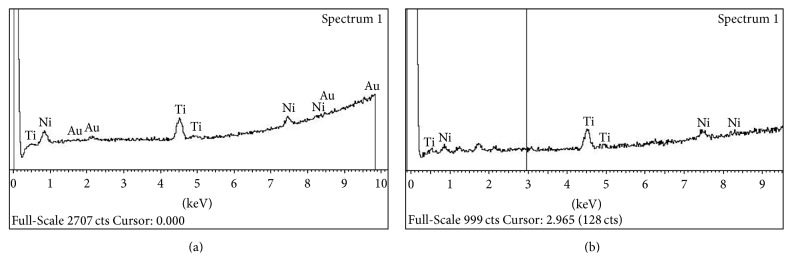
EDS analysis of as-received K3 instrument (a) and HF-treated K3 instrument (b). As-received and HF-treated K3 instruments showed no difference in ion composition.

As-received and HF-treated K3 file had no difference in NiTi composition. The results were illustrated in Table 2 and Figure 3.

### 3.4. Examination of Fracture Surface

**Figure 4:**
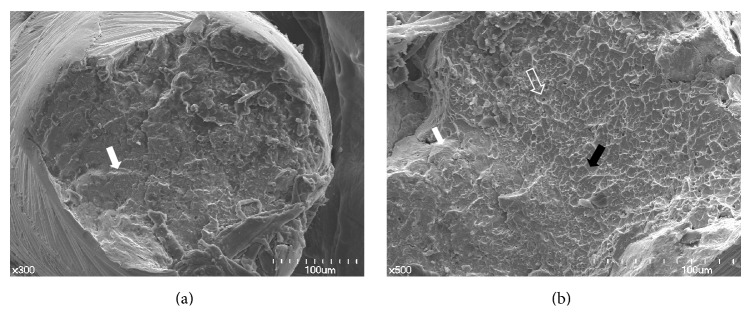
Fracture surface of as-received (a) and HF-treated (b) K3 instruments. Both surfaces revealed characteristic patterns of cyclic fatigue fractures with multiple lines (white arrows in (a, b)) and cone and cup appearance (black arrows in (b)). Pore-like structures were also shown (unfilled white arrow in (b)). The multiple lines pointed to by white arrow show the initiation site for the fatigue fracture process.

Figure 4(a) (as-received K3 instrument) and Figure 4(b) (HF-treated K3 instrument) show the fracture surface of each group of K3 NiTi instruments. Figure 4(a) shows the multiple lines (white arrow) and cone and cup appearance at the same time. Figure 4(b) also shows the multiple lines (white arrow), dimple (black arrow) appearance, and pore-like structures (unfilled white arrow). Multiple lines correspond to the advance of the crack front in successive cycles.

## 4. Discussion

There are several kinds of surface treatments to improve surface characteristics of ground NiTi rotary instrument. Among these, electropolishing has been relatively much investigated. However, there is a still controversy regarding the effectiveness of electropolishing on cyclic fatigue resistance of NiTi rotary files. Some studies [19–21] demonstrated the positive effect of electropolishing on the fatigue resistance of NiTi rotary files, while other studies [22] did not. This was similar to the case of ion implantation. Gavini et al. [23] reported that the nitrogen ion implantation was effective in increasing the fatigue resistance of NiTi rotary instrument. On the other hand, Wolle et al. [14] reported that argon implantation reduced the fatigue resistance of NiTi rotary instrument.

However, until now, no study has been published on the effect of simple treatment of strong acid on the cyclic fatigue resistance of NiTi rotary instruments. This is the first study that investigated the effect of concentrated HF treatment on the surface smoothness and the fatigue resistance of NiTi rotary instrument.

HF is a component of Kroll's reagent, which is used for chemical polishing of manufactured NiTi rotary instruments [18]. In this experiment, higher concentration (50%) than that of HF in Kroll's reagent was used and two main actions were postulated with this acid treatment. Firstly, as was supported by SEM examination results obtained with K3 instruments, HF surface treatment reduced the rough surface and stress concentrating microdefect, which might improve the cyclic fatigue resistance in K3 instruments. This is in agreement with result of Karn et al. [24]. Secondly, as was suggested by a previous study [25], HF surface treatment might anodize and passivate the titanium oxide layer which was produced during manufacturing, which might also improve cyclic fatigue resistance. The smoothening of rough surfaces of NiTi rotary files could give additional benefit which is related to biocompatibility. The rougher surface and larger surface area tend to have more corrosion than smoother and smaller surface area. The surface corrosion of NiTi alloy could release nickel ion that can evoke allergic reactions [26] in contacting tissues. In this sense, HF treatment which resulted in smoother surface in this experiment is postulated to contribute to increasing the cyclic fatigue resistance of NiTi files as well as lowering the corrosion tendency of them. In this sense, future investigations on the corrosion resistance of HF-treated NiTi instruments are needed.

“Kroll's reagent,” the mixture of HF and nitric acid in water, has been widely used as chemical etching agent for alloys used for hip and knee prosthesis [27] and dental implant [28, 29]. “Kroll's reagent” is listed as ASTM (American Society of Testing Materials) #187 etchant which was recommended to be used for microchemical etching of cast irons. Usually, the relative composition of HF in Kroll's solution is approximately 5%. However, in this experiment, 10 times higher concentrations of HF were used for chemical etching, because our preliminary study showed that high concentrations of HF were effective in increasing the fatigue resistance of K3 NiTi files (unpublished data).

The composition of Ni and Ti in NiTi rotary files has been known to be 55 : 45 and the composition of Ni and Ti has been known to affect the mechanical properties of NiTi alloy [30]. The present study showed that the HF treatment did not affect the Ni and Ti composition. Further studies regarding the effect of various surface treatments or corrosion of NiTi rotary instruments on the Ni and Ti composition would be necessary.

This study has several limitations, one of which was that the minute surface changes (e.g., formation of surface oxide) could not be thoroughly investigated; the other is that the investigation of minute surface changes in instruments could be better investigated with more precise devices. A surface sensitive technique (e.g., X-ray photoelectron spectroscopy and Auger Spectroscopy) would be more useful to understand the chemical changes of the surface after HF etching (e.g., changes of Ni/Ti ratio, increase of oxygen concentration, and formation of passivation layers). So the future study using more precise equipment than EDS to investigate exact Ni and Ti ratio might be necessary. Future studies regarding the effect of HF on cutting efficiency might be also necessary. Moreover, future studies on the effect of HF concentration and duration of the treatment on surface characteristics and resistance to fatigue fracture might be helpful to better understand the role of HF treatment.

In conclusion, this study clearly demonstrated that HF surface treatment could be beneficial in improving the surface smoothness and thus increasing the cyclic fatigue resistance of ground NiTi rotary files.

## Figures and Tables

**Table 1:** The results of fatigue resistance test of HF-treated and as-received K3 instruments.

NCF	K3 (*n* = 20)
As-received	79825.5 (±14472.35)^a^
HF-treated	90835.5 (±13170.87)^b^

NCF: number of cycles to fracture; HF: hydrofluoric acid. Different alphabetical letter represents statistically significant difference.

**Table 2:** Ni and Ti composition in as-received and HF-treated samples in K3 (atomic%).

Weight percent	Ni	Ti
As-received (*n* = 3)	55.07 ± 2.50	44.93 ± 2.50
HF-treated (*n* = 3)	54.14 ± 2.70	45.86 ± 2.70
